# Clinical outcomes and predictive modeling in COVID-19 patients with type 2 diabetes mellitus: a multicenter retrospective cohort study

**DOI:** 10.1186/s12879-025-11578-y

**Published:** 2025-10-21

**Authors:** Kaiheng Guo, Haini Zhi, Xiaoying Zhou, Shaofeng Huang, Junxu Lin, Jinxin Pang, Lu Xiao, Weiping Sun, Chunping Zeng

**Affiliations:** 1 Department of Endocrinology and Metabolism, The Fifth Affiliated Hospital of Guangzhou Medical University, No.621, Gangwan Road, Guangzhou, 540700 China; 2 Department of Endocrinology and Metabolism, Loudi Central Hospital, No. 51 Changqing Street, Louxing District, Loudi, 417099 China

**Keywords:** Type 2 diabetes mellitus, COVID-19, Multicenter studies, Models, statistical

## Abstract

**Background:**

This study aimed to assess the influence of type 2 diabetes mellitus (T2DM) on clinical features and adverse outcomes in COVID-19 patients and to develop a predictive model for adverse outcomes in this population.

**Methods:**

A retrospective analysis was conducted from December 2022 to February 2023, involving 1058 COVID-19 inpatients at two hospitals. Patients were divided into T2DM (*n* = 363) and non-T2DM (*n* = 695) groups. Demographic and laboratory data were collected, and univariate analyses were performed. Logistic regression analysis was employed to identify risk factors associated with ICU admission, and a predictive model was constructed and validated using ROC curves.

**Results:**

T2DM patients exhibited higher levels of certain inflammatory and biochemical markers and a greater incidence of ICU admission compared to non-T2DM patients. Neutrophil count and lactate dehydrogenase were identified as independent risk factors for ICU admission.

**Conclusions:**

T2DM is associated with increased levels of inflammatory and biochemical markers and a higher risk of ICU admission in COVID-19 patients. The predictive model, incorporating neutrophil count and lactate dehydrogenase, offers clinical utility. The study’s findings can inform clinical strategies for managing COVID-19 patients with T2DM, particularly in predicting and mitigating adverse outcomes.

## Introduction

The coronavirus disease 2019 (COVID-19) caused by the severe acute respiratory syndrome (SARS) coronavirus 2 (SARS-CoV-2) has become a rapidly spreading infectious disease globally since 2019, with significant impact on human health and economic burden [1]. The Chinese Center for Disease Control and Prevention (CDC) reported a peak of 6.94 million COVID-19 nucleic acid-positive cases and a peak of 1.625 million hospitalizations since the full lifting of controls on COVID-19 in December 2022 in mainland China. As of today, COVID-19 is still spreading globally.

Diabetes mellitus (DM) is a risk factor for COVID-19 rapid progression and poor prognosis among common comorbidities [2, 3]. Moreover, DM is a pro-inflammatory disease that is typically accompanied by immune dysfunction [4]. As a result, patients with DM may experience a state of excessive inflammation and immunosuppression when infected with SARS-CoV-2. The clinical presentation of COVID-19 is nonspecific, ranging from asymptomatic to respiratory symptoms including fever, cough, and dyspnea, and in severe cases can progress to adverse outcomes such as respiratory failure, intensive care unit (ICU) transfer, and even death [5]. Some studies have shown that higher blood glucose levels, both short- and long-term, are associated with worse COVID-19 outcomes, including longer hospital stays, increased need for mechanical ventilation and ICU admissions, and higher mortality [6, 7]. One study pointed out that DM increases the risk of ICU admission in patients with COVID-19, with 38.8% of DM patients admitted to the ICU compared to 10.06% in the non-DM group [8]. However, studies on risk factors and prediction of ICU admission for COVID-19 patients with diabetes are still scarce.

Based on the above questions, we launched the following two-part study. First, we compared the clinical characteristics of the two groups of type 2 diabetes mellitus (T2DM) and non-T2DM among COVID-19 patients, and the incidence of their adverse outcomes (admission to the ICU, respiratory failure, endotracheal intubation, and death) by means of a retrospective cohort study. Second, based on the statistically different clinical adverse outcomes (admission to ICU) derived in the first part, we explored the risk factors for transfer to ICU in patients with COVID-19 combined with T2DM and constructed its prediction model. It aims to analyze the impact of T2DM on COVID-19 patients and to explore the relationship between T2DM and COVID-19 in terms of clinical examination indicators, as well as the correlation between the occurrence of adverse outcomes in COVID-19 patients with T2DM. In addition, risk factors for adverse outcomes were identified and predictive models were developed.

## Methods

### Study design and patients

This retrospective multicenter study collected 1058 patients with confirmed COVID-19 with or without T2DM who were hospitalized at The Fifth Affiliated Hospital of Guangzhou Medical University and Loudi Central Hospital in China from December 1, 2022 to February 1, 2023. The study was conducted in compliance with the Declaration of Helsinki and approved by the Research Ethics Committee of the Fifth Affiliated Guangzhou Medical University (GYWY-L2023-64). Individual consent was not required because all data were anonymized before analysis.

Inclusion criteria were (1) age ≥ 18 years; and (2) with one or more of the following pathogenetic and serologic findings: a positive SARS-CoV-2 nucleic acid test; a positive SARS-CoV-2 antigen test; and a positive SARS-CoV-2 isolation and culture; or SARS-CoV-2 specific IgG antibody level is 4 times or more elevated in the recovery phase than in the acute phase.

Exclusion criteria were (1) age < 18 years; (2) patients who did not meet the diagnostic criteria for SARS-CoV-2 infection; (3) patients who were pregnant, had a history of active malignancy, or had a glomerular filtration rate of < 15 ml/min/1.73 m^2^; (4) patients with severe psychiatric disorders. (5) patients with serious data deficiencies in the electronic medical record system; and (6) patients with nosocomial SARS-CoV-2 infection.

In the first phase, patients diagnosed with T2DM before or at the time of admission to the hospital were considered as the experimental group, and the diagnosis of T2DM was based on the American Diabetes Association (ADA) diabetes guidelines [9], while the other patients were considered as the control group. We collected baseline information, laboratory results, and outcome information for each patient through the electronic medical record. The outcome information included whether or not the patient was admitted to the ICU, experienced respiratory failure, required tracheal intubation, or died. All collected data were reviewed by research members and double-checked by experienced physicians. In the second stage, for further in-depth analysis of patients with COVID-19 combined with T2DM, we divided these patients into ICU-admitted and non-ICU-admitted groups. The influencing factors of COVID-19 combined T2DM patients admitted to ICU were analyzed and their prediction models were constructed.

### Statistical analysis

All data were analyzed using the IBM SPSS Statistics V26.0 (IBM Corp.: Armonk, NY, USA). Quantitative data were expressed as x ± s. If differences between groups were normally distributed, they were compared using the independent samples t-test, whereas quantitative and ordinal data that did not conform to a normal distribution were expressed as median or interquartile ranges and differences between groups were compared using nonparametric tests. Qualitative data were described by frequencies or percentages, and differences between groups were compared using the X2 test. For all statistical analyses, a p value of < 0.05 was considered statistically significant. On the other hand, the influencing factors with statistically significant differences were analyzed using multifactor logistic regression, and the predictive model was constructed based on the results of the multifactor analysis, and its goodness of fit was tested using the Hosmer-Lemeshow test. Afterwards, the predictive value of the prediction model was analyzed using the receiver operating characteristic (ROC) curve with a test level = 0.05. To quantify potential optimistic bias in model performance and provide more accurate estimates of predictive accuracy, this study employed bootstrap resampling for internal validation. Optimistic bias was calculated for each iteration using the formula “optimistic bias = Area Under the Curve (AUC) training set - AUC test set”, and the final corrected AUC was obtained by subtracting the average optimistic bias from the original AUC. It was performed in the R environment (version 4.5.1) using the pROC and boot packages.

## Results

**Fig. 1:**
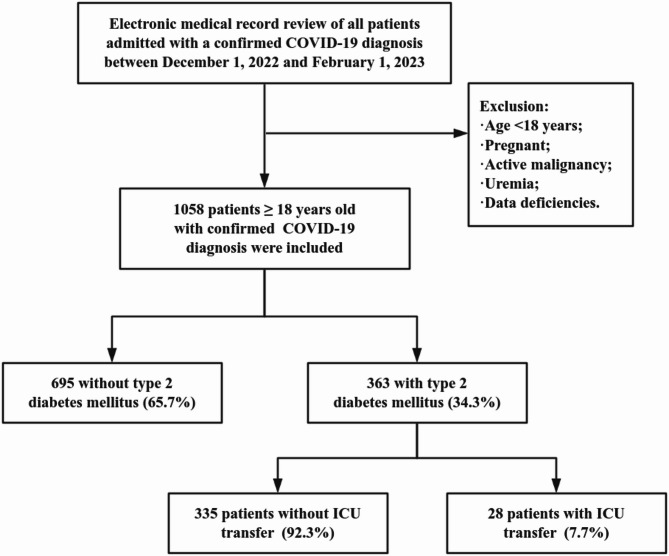
Patient selection flow chart

A total of 1058 patients with COVID-19 were enrolled in this retrospective study, including 363 T2DM patients (34.3%) and 695 non-T2DM patients (65.7%). The patient selection flow chart is shown in Fig. 1.

### Baseline and clinical characteristics of COVID-19 patients

The baseline characteristics of the admitted patients are shown in Table 1. Among all hospitalized patients, there were no significant differences in age and gender between the two groups. In terms of vital signs at admission, patients with T2DM had higher systolic blood pressure compared with non-T2DM patients (132 [IQR, 120–146] vs. 128 [IQR, 114–140], *p* < 0.01), whereas there were no significant differences in diastolic blood pressure and heart rate (both *p* > 0.05). As for the comorbidities of other chronic diseases, the proportion of patients with hypertension (214 [59.0%] vs. 264 [38.0%], *p* < 0.01), coronary heart disease (128 [35.3%] vs. 174 [25.0%], *p* < 0.01), and chronic kidney disease (28 [7.7%] vs. 13 [1.9%], *p* < 0.01) was higher in the T2DM group than that of the non-T2DM group, while the proportion of patients with chronic bronchial disease and cerebrovascular disease was not significantly different between the two groups (both *p* > 0.05).

**Table 1:** Comparison of baseline characteristics between non-T2DM and T2DM in COVID-19 patients

	Median (IQR) or *n* (%)			*P* value
	Total(*n* = 1058)	non-T2DM(*n* = 695)	T2DM(*n* = 363)	
Age (years)	71.5(59–80)	71(59–81)	72(62–80)	0.974
Gender (male)	612(57.8%)	392(56.4%)	220(60.6%)	0.189
Systolic blood pressure (mm Hg)	130(116–143)	128(114–140)	132(120–146)	0.001*
Diastolic blood pressure (mm Hg)	77(70–85)	77(70–85)	76(70–85)	0.736
Heart rate (bpm)	88(78–99)	88(77–99)	88(80–100)	0.246
Comorbidities on Admission				
Hypertension	478(45.2%)	264(38.0%)	214(59.0%)	0.000*
Coronary heart disease	302(28.55)	174(25.0%)	128(35.3%)	0.000 *
Chronic bronchial diseases	165(15.65)	114(16.4%)	51(14.0%)	0.317
Cerebrovascular disease	117(11.1%)	71(10.2%)	46(12.7%)	0.226
Chronic renal diseases	41(3.9%)	13(1.9%)	28(7.7%)	0.000*

The presence of an asterisk (*) indicates statistical significance at *p* < 0.05

Differences in laboratory results between T2DM and non-T2DM patients with COVID-19 are shown in Table 2. All patients had C-reactive protein (CRP), interleukin-6 (IL-6), procalcitonin (PCT), creatine kinase isoenzyme (CK-MB), fibrinogen (Fib), and D-dimer above the normal range, and lymphocyte count (Lymph), serum albumin (ALB) below the normal range, and the rest of the indices were in the normal range. In T2DM patients, compared to the non-T2DM group, IL-6 (21.64 [IQR, 5.45–74.65] vs. 12.85 [IQR, 3.70-74.65], *p* < 0.01), white blood cell count (WBC) (6.75 [IQR, 5.06–9.42] vs. 6.39 [IQR, 4.57–8.68], *p* = 0.036), neutrophil count (Neut) (4.96 [IQR, 3.30–7.66] vs. 4.52 [IQR, 3.08–6.75], *p* < 0.01), creatinine (Crea) (84.00 [IQR, 65.00-109.00] vs. 72.00 [IQR, 58.00-92.80], *p* < 0.01), CK-MB (14.00 [IQR, 10.00–19.00] vs. 13.00 [IQR, 9.00–17.00], *p* < 0.01), serum potassium (K^+^) (4.22 [IQR, 3.75–4.59] vs. 4.00 [IQR, 3.60–4.42], *p* < 0.01), and Fib (4.72 [IQR, 3.64–5.80] vs. 4.45 [IQR, 3.38–5.90], *p* = 0.048) levels were elevated. While, ALB (34.10 [IQR, 30.30–36.40] vs. 35.10 [IQR, 31.60–38.10], *p* < 0.01), serum sodium (Na^+^) (136.66 [IQR, 134.02-138.92] vs. 137.27 [IQR, 135.27-139.98], *p* < 0.01), prothrombin time (PT) (11.80 [IQR, 11.10–12.40] vs. 11.90 [IQR, 11.20–12.60], *p* < 0.01), and activated partial thromboplastin time (APTT) (30.10 [IQR, 25.60–34.20] vs. 31.30 [IQR, 27.30–35.50]. *p* < 0.01) was significantly lower.

**Table 2:** Comparison of laboratory data for non-T2DM vs. T2DM in COVID-19 patients

	Normal range	Median (IQR)		*P* value
		non-T2DM(*n* = 695)	T2DM(*n* = 363)	
CRP(mg/L)	0–10	42.72(8.30-49.23)	42.72(9.28–59.04)	0.053
IL-6(pg/ml)	0–7	12.85(3.70-74.65)	21.64(5.45–74.65)	0.001*
PCT(ng/ml)	0-0.05	0.10(0.04–1.35)	0.12(0.05–0.86)	0.443
WBC(×109/L)	3.5–9.5	6.39(4.57–8.68)	6.75(5.06–9.42)	0.036*
Neut(×109/L)	1.8–6.3	4.52(3.08–6.75)	4.96(3.30–7.66)	0.008*
Lymph(×109/L)	1.1–3.2	0.98(0.64–1.40)	0.94(0.62–1.32)	0.646
PLT(×109/L)	125–350	200.00(148.00-264.00)	195.00(148.00-254.00)	0.448
HGB(g/L)	115–150	126.00(114.00-138.00)	126.00(113.00-139.00)	0.897
Crea(umol/L)	41–81	72.00(58.00-92.80)	84.00(65.00-109.00)	0.000*
ALT(U/L)	7–40	21.00(14.91-32.00)	21.00(14.30–31.00)	0.720
AST(U/L)	13–35	30.00(21.00-40.96)	27.03(19.00–38.00)	0.001*
TBIL(umol/L)	0–23	9.23(6.80–12.80)	9.10(6.52–11.80)	0.079
DBIL(umol/L)	0–8	2.80(0.00-4.20)	2.80(0.00–4.00)	0.864
CK-MB(ng/ml)	0–5	13.00(9.00–17.00)	14.00(10.00–19.00)	0.009*
LDH(U/L)	120–250	215.00(178.00-268.00)	218.00(183.00-276.00)	0.451
ALB(g/L)	40–55	35.10(31.60–38.10)	34.10(30.30–36.40)	0.001*
K+(mmol/L)	3.5–5.3	4.00(3.60–4.42)	4.22(3.75–4.59)	0.000*
Na+(mmol/L)	137–147	137.27(135.27-139.98)	136.66(134.02-138.92)	0.000*
PT(s)	9.8–13.5	11.90(11.20–12.60)	11.80(11.10–12.40)	0.002*
APTT(s)	22.5–34	31.30(27.30–35.50)	30.10(25.60–34.20)	0.000*
Fib(g/L)	1.8-4	4.45(3.38–5.90)	4.72(3.64–5.80)	0.048*
D-Dimer(mg/L)	<0.55	0.71(0.30–1.56)	0.72(0.33–1.56)	0.746

*CRP* C-reactive protein, *IL-6* interleukin-6, *PCT* procalcitonin, *WBC* white blood cell count, *Neut *neutrophil count, *Lymph* lymphocyte count, *PLT* blood platelet, *HGB *hemoglobin, *Crea* creatinine, *ALT* alanine aminotransferase, *AST* aspartate aminotransferase, *TBIL* total bilirubin, *DBIL *direct bilirubin, *CK-MB* creatine kinase isoenzyme, *LDH* lactate dehydrogenase, *ALB* albumin, *K+* potassium, *Na+* sodium, *PT* prothrombin time, *APTT* activated partial thromboplastin time, *Fib* fibrinogen

The presence of an asterisk (*) indicates statistical significance at *p* < 0.05

### Comparison of adverse outcomes in COVID-19 patients with and without T2DM

The distribution of adverse outcomes in patients with COVID-19 by whether or not they had T2DM is presented in Table 3. Patients with T2DM demonstrated a higher incidence of ICU admission (28 [7.75%] vs. 18 [2.6%], *p* < 0.01), whereas there was no significant difference between the two groups in terms of the incidence of respiratory failure as well as tracheal intubation, and mortality.

**Table 3:** Comparison of adverse outcomes in COVID-19 patients with non-T2DM versus T2DM

	*n* (%)			*P* value
	Total(*n* = 1058)	non-T2DM(*n* = 695)	T2DM(*n* = 363)	
Respiratory failure	305(28.8%)	198(28.5%)	107(29.5%)	0.736
Tracheal intubation	56(5.3%)	34(4.9%)	22(6.1%)	0.420
Admission to ICU	46(4.3%)	18(2.6%)	28(7.75%)	0.000*
Mortality	39(3.7%)	26(3.7%)	13(3.6%)	0.896

The presence of an asterisk (*) indicates statistical significance at *p* < 0.05

### Identification of significant predictors of ICU admission in patients of COVID-19 combined with T2DM

Table 4 presents the comparison of clinical characteristics between ICU-admitted and non-ICU-admitted patients with COVID-19 combined T2DM. The results of univariate analysis showed that the differences between the ICU-admitted and non-ICU-admitted groups were statistically significant in terms of CRP, IL-6, PCT, WBC, Neut, AST, CK-MB, LDH, ALB, PT, and APTT (*p* < 0.05). The above statistically significant variables were included in binary logistic regression analysis and the results of logistic regression analysis are shown in Table 5. The variance inflation factors (VIF) of all predictor variables were significantly lower than the critical threshold (VIF < 5), indicating that there was no significant multicollinearity interference between variables and that the model parameter estimates were reliable. The final results suggested that Neut (OR = 1.018, 95% CI 1.002–1.034, *p* = 0.028) and LDH (OR = 1.007, 95% CI 1.003–1.012, *p* = 0.001) were the independent risk factors for ICU admission in patients with COVID-19 combined T2DM.

**Table 4:** The factors influencing ICU admission of patients with COVID-19 combined with T2DM

	Median (IQR) or *n* (%)			*P* value
	Total(*n* = 363)	non-ICU-admitted(*n* = 335)	ICU-admitted(*n* = 28)	
Age (years)	72(62–80)	72(62–80)	70(64.25-77)	0.428
Systolic blood pressure (mm Hg)	132(120–146)	132(120–146)	136(119.25–148)	0.542
Systolic blood pressure (mm Hg)	76(70–85)	76(70–85)	79.5(69.5–90)	0.136
Heart rate (bpm)	88(80–100)	88(79–100)	87(80.5-108.75)	0.512
Gender (male)	220(60.6%)	201(60.0%)	19(67.9%)	0.414
Comorbidities on Admission				
Hypertension	214(59.0%)	196(58.5%)	18(64.3%)	0.550
Coronary heart disease	128(35.3%)	115(34.3%)	13(46.4%)	0.198
Chronic bronchial diseases	51(14.0%)	48(14.3%)	3(10.7%)	0.781
Cerebrovascular disease	46(12.7%)	39(11.6%)	7(25.0%)	0.068
Chronic renal diseases	28(7.7%)	24(7.2%)	4(14.3%)	0.255
laboratory data				
CRP(mg/L)	42.72(9.28–59.04)	42.72(9.03–56.06)	43.01(30.15–81.36)	0.044*
IL-6(pg/ml)	21.64(5.45–74.65)	20.05(4.92–74.65)	44.48(13.52–98.95)	0.019*
PCT(ng/ml)	0.12(0.05–0.86)	0.10(0.05–0.72)	0.38(0.14–1.23)	0.006*
WBC(×109/L)	6.75(5.06–9.42)	6.64(4.91–8.83)	8.84(5.65–13.49)	0.006*
Neut(×109/L)	4.96(3.3–7.66)	4.88(3.28-7.00)	7.66(4.52–13.31)	0.002*
Lymph(×109/L)	0.94(0.62–1.32)	0.95(0.64–1.32)	0.82(0.46–1.52)	0.221
PLT(×109/L)	195(148–254)	195.00(149.00-254.00)	185.50(129.75-248.75)	0.518
HGB(g/L)	126(113–139)	126.00(113.00-139.00)	123.50(108.75-142.25)	0.603
Crea(umol/L)	84(65–109)	83.00(65.00-106.88)	100.53(65.45–133.00)	0.213
ALT(U/L)	21(14.3–31)	21.00(14.30–30.00)	21.67(14.25-38.00)	0.528
AST(U/L)	27.03(19–38)	26.92(18.70-37.98)	35.11(24.48–50.29)	0.009*
TBIL(umol/L)	9.1(6.52–11.8)	9.10(6.60–11.90)	8.95(5.23–11.26)	0.275
DBIL(umol/L)	2.8(0–4)	2.90(0.00–4.00)	2.35(1.55–4.30)	0.788
CK-MB(ng/ml)	14(10–19)	14.00(10.00–18.00)	17.50(11.08–37.50)	0.023*
LDH(U/L)	218(183–276)	207.00(179.00-262.00)	294.00(248.25-419.25)	0.000*
ALB(g/L)	34.1(30.3–36.4)	34.30(31.00-36.60)	29.85(28.00-34.90)	0.007*
K+(mmol/L)	4.22(3.75–4.59)	4.22(3.75–4.59)	4.22(3.74–4.99)	0.532
Na+(mmol/L)	136.66(134.02-138.92)	136.62(134.05-138.87)	136.80(130.71-139.67)	0.795
PT(s)	11.8(11.1–12.4)	11.7(11.1–12.4)	12.15(11.43–13.08)	0.028*
APTT(s)	30.1(25.6–34.2)	29.70(25.20–33.60)	33.70(28.65–37.03)	0.006*
FiB(g/L)	4.72(3.64–5.8)	4.72(3.70–5.80)	4.74(3.23–6.28)	0.951
D-Dimer(mg/L)	0.72(0.33–1.56)	0.70(0.33–1.56)	1.19(0.36–1.60)	0.276

*CRP* C-reactive protein, *IL-6* interleukin-6, *PCT* procalcitonin, *WBC* white blood cell count, *Neut* neutrophil count, *Lymph *lymphocyte count, *PLT* blood platelet, *HGB* hemoglobin, *Crea* creatinine, *ALT* alanine aminotransferase, *AST *aspartate aminotransferase, *TBIL* total bilirubin, *DBIL* direct bilirubin, *CK-MB *creatine kinase isoenzyme, *LDH *lactate dehydrogenase, *ALB* albumin, *K+* potassium, *Na+* sodium, *PT* prothrombin time, *APTT* activated partial thromboplastin time, *Fib* fibrinogen

The presence of an asterisk (*) indicates statistical significance at *p* < 0.05

**Table 5:** Multivariate logistic regression analysis to detect predictors of ICU admission in COVID-19 patients with T2DM

	Odds Ratio (OR)	95% Confidence Interval	*P* value
CRP	0.995	0.984–1.006	0.367
IL-6	1	1.000-1.001	0.583
PCT	1.017	0.905–1.143	0.772
WBC	1.003	0.937–1.073	0.937
Neut	1.018	1.002–1.034	0.028*
AST	0.991	0.981–1.002	0.105
CK-MB	1.002	0.985–1.019	0.807
LDH	1.007	1.003–1.012	0.001*
ALB	0.976	0.904–1.054	0.541
PT	1.061	0.856–1.315	0.591
APTT	1.019	0.978–1.061	0.373

*CRP* C-reactive protein, *IL-6 *interleukin-6, *PCT* procalcitonin, *WBC* white blood cell count, *Neut* neutrophil count, *AST* aspartate aminotransferase, *CK-MB* creatine kinase isoenzyme, *LDH* lactate dehydrogenase, *ALB* albumin, *PT* prothrombin time, *APTT* activated partial thromboplastin time

The presence of an asterisk (*) indicates statistical significance at *p* < 0.05

### Construction and validity evaluation of a prediction model for ICU admission of patients with COVID-19 combined T2DM

**Fig. 2:**
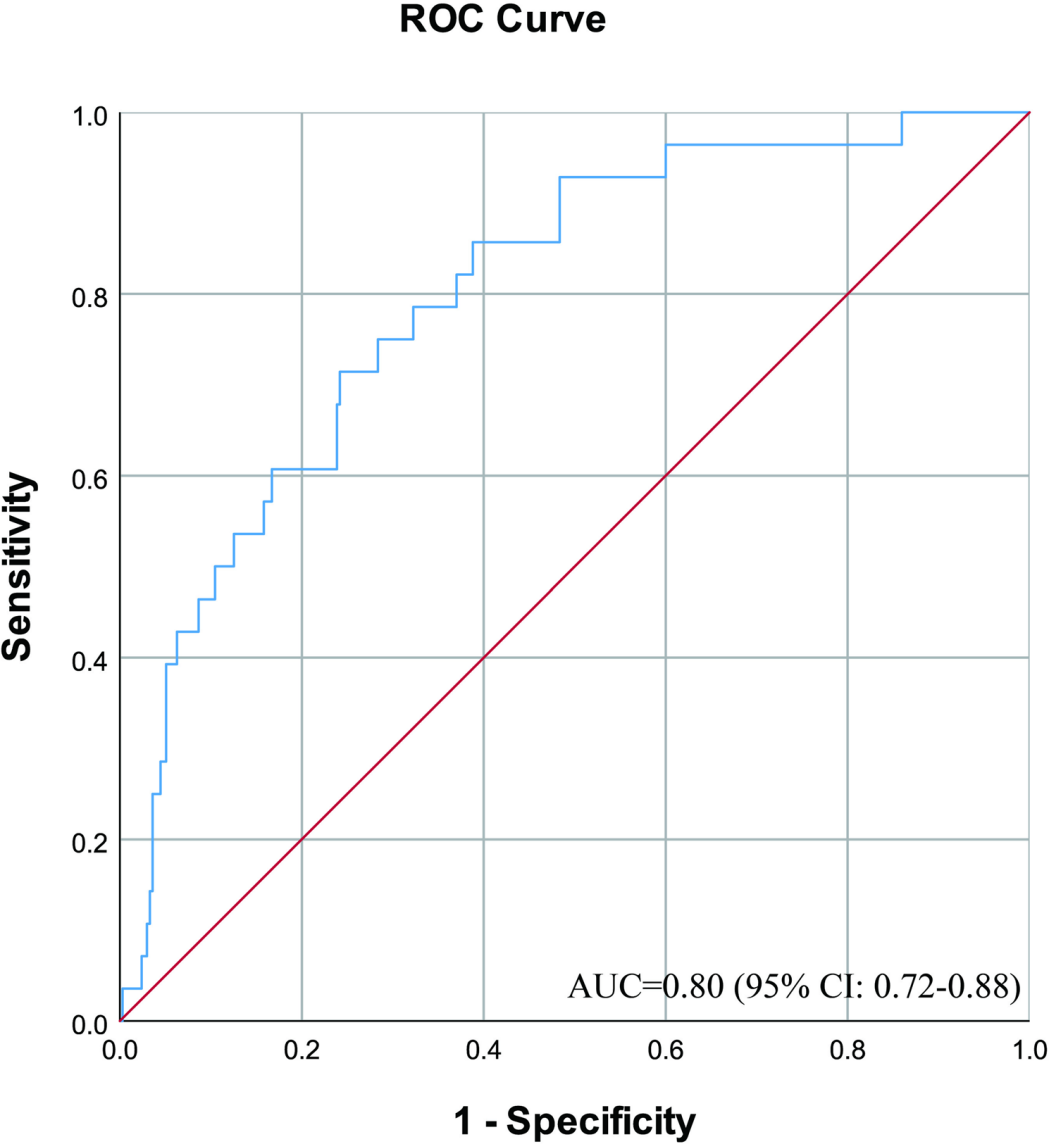
ROC curves from a risk prediction model for ICU admission of patients with COVID-19 combined T2DM

**Fig. 3:**
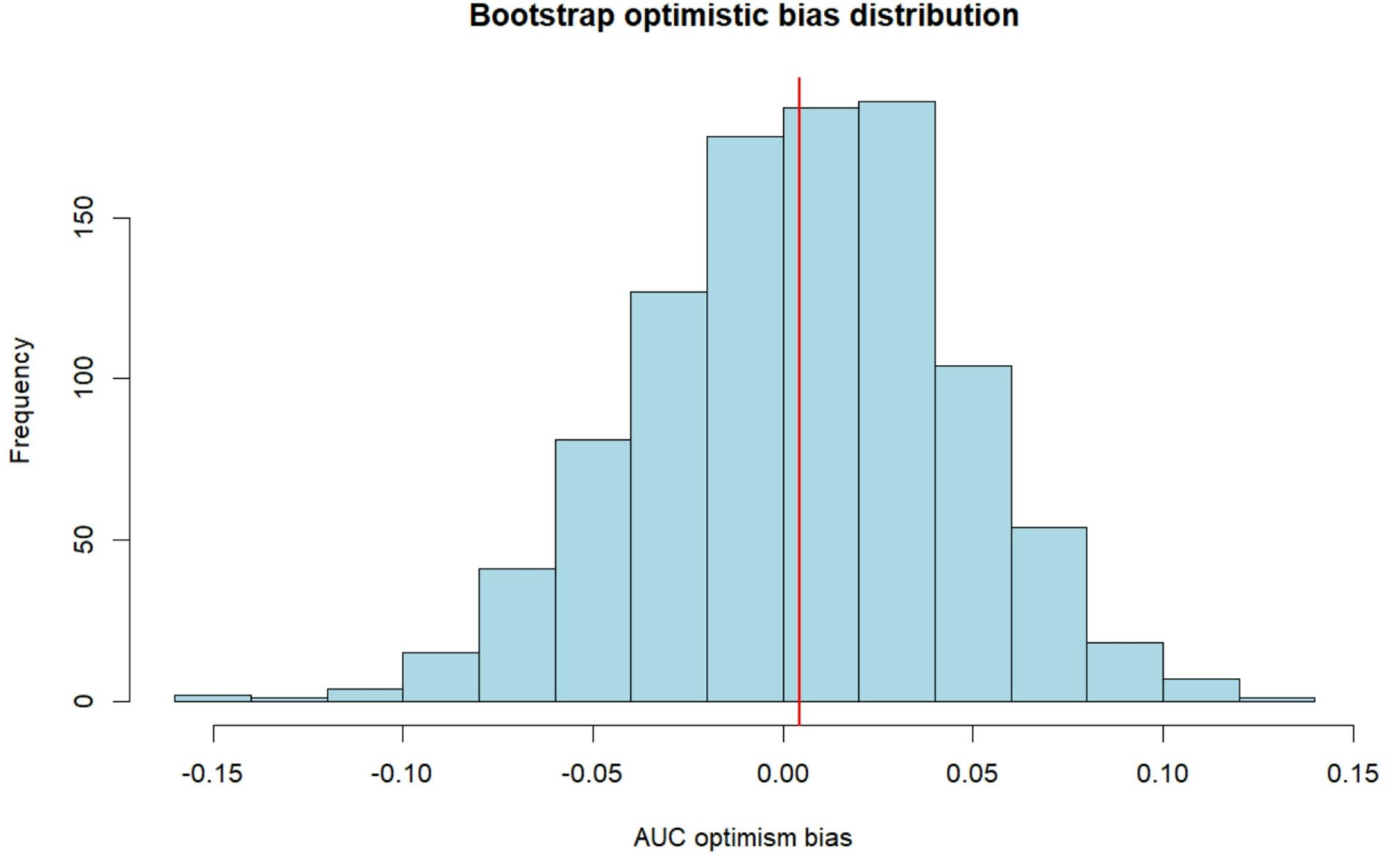
Bootstrap optimism bias distribution for AUC

The risk prediction equation was logit(P) = −3.162 + 0.016 × Neut + 0.002 × LDH. The Hosmer-Lemeshow test p-value was 0.547 (*p* > 0.05), suggesting that the predictive model had a good fit. The established model was validated by plotting the ROC curve (Fig. 2), which resulted in an AUC of 0.80 (95% CI: 0.72–0.88), indicating that the constructed risk prediction model had good discriminatory power. Internal validation using Bootstrap (1,000 iterations) demonstrated excellent model stability. The original apparent AUC was 0.799 (95% CI: 0.72–0.88), with an average optimistic bias of 0.004 (95% CI: −0.078 to 0.080), and the adjusted AUC was 0.795. As shown in Fig. 3, the optimistic bias exhibits a symmetric distribution centered at zero, indicating that the model poses minimal risk of overfitting. The adjusted calibration slope is 0.98 (95% CI: 0.92–1.05), further confirming that the model maintains good calibration accuracy.

## Discussion

This is a multicenter retrospective study to evaluate and compare the demographic, clinical characteristics and adverse outcomes of SARS-CoV-2 infections in patients with T2DM versus non-T2DM patients. We found that patients in the T2DM group had higher systolic blood pressure and higher rates of underlying diseases such as hypertension, coronary artery disease, and history of chronic kidney disease than the control group. Diabetic COVID-19 patients had elevated levels of IL-6, WBC, NEUT, Crea, CK-MB, K+, and Fib, while ALB, Na+, PT, and APTT were decreased. Moreover, T2DM patients were at higher risk of ICU admission.

From these results, it is clear that the majority of patients hospitalized for COVID-19 are seniors, who are at a higher threat of contracting COVID-19 due to their weakened immunity and multiple associated comorbidities such as diabetes mellitus, hypertension, coronary artery disease (CAD), chronic kidney disease (CKD), and chronic obstructive pulmonary disease (COPD) [10]. In addition, patients with diabetes mellitus (DM) are more likely to have a combination of hypertension, CAD, and CKD than normal subjects. DM causes hypertension through atherosclerosis and plaque formation, as well as increases the risk of CAD through the deposition of glycosylation end-products in the endothelial cells of coronary arteries, and contributes to the development of CKD through the impact of DM on the filtration function of the kidneys through microvascular pathology [11]. It is in line with the results of the study we obtained. In contrast, several studies have shown that the combination of COVID-19 with DM and other diseases above increases the risk of adverse outcomes and death [12, 13].

DM involves immune dysfunction, endothelial dysfunction, and pro-inflammatory cytokine storm, all of which exacerbate COVID-19 susceptibility and risk of adverse outcomes [14]. Hyperglycemia enhances SARS-CoV-2 replication and pro-inflammatory cytokine production in monocytes through high expression of angiotensin-converting enzyme 2 (ACE2), resulting in higher inflammatory markers in COVID-19 patients [15]. Several studies have found that diabetic patients infected with SARS-CoV-2 exhibit manifestations of excessive inflammation and T-cell dysfunction, and that COVID-19 diabetic patients have significantly elevated levels of IL-6, leading to a pro-inflammatory state that may promote systemic inflammation in the patient [2, 16]. Thus, the pro-inflammatory state associated with diabetes may contribute to the poor prognosis of COVID-19 patients. In addition, similar to the findings of one study [17], COVID-19 patients with T2DM were more likely to have low-protein malnutrition, which was associated with increased cytokine expression and impaired immune function.

DM is a chronic metabolic disorder characterized by high blood glucose levels, and ACE2 expression is elevated in T2DM patients. Then SARS-CoV-2 binds to ACE2 receptor, which can lead to multiple organ damage such as heart and kidney [14, 18, 19]. We found that creatinine was significantly elevated in patients with comorbid T2DM, suggesting that diabetes may be an aggravating factor for COVID-19 kidney damage. According to a meta-analysis [20], the etiology of COVID-19 renal impairment may be multifactorial, and in addition to direct attack by SARS-CoV-2, hypoxia and hypercoagulability can also lead to renal injury. While hyperglycemia leads to high ACE2 expression, binding of ACE2 receptors to SARS-CoV-2 exacerbates immune deficits in T2DM patients and leads to a pro-inflammatory and pro-coagulant state, resulting in endothelial dysfunction, oxidative stress, and microvascular injury, all of which worsen renal function [21, 22]. Moreover, the deterioration of renal function will be more pronounced if this patient is combined with diabetic nephropathy. In addition, SARS-CoV-2 causes cardiac damage through viral cell invasion and active viral replication in cardiomyocytes, causing myocarditis and advanced fibrosis. An experimental study confirms that ACE2 expression in cardiac tissue is increased in diabetes and promotes greater cellular entry of SARS-CoV-2, leading to a worse prognosis in these patients [23]. Similar to one study [24], COVID-19 patients with T2DM had higher troponin-T, CK-MB levels compared to those without T2DM, indicating that diabetes exacerbates the clinical outcome of COVID-19.

Pathophysiologically, both DM and SARS-CoV-2 induce inflammation and oxidative stress with sustained damage to the endothelium, which may lead to deterioration of one or the other. Hypercoagulability and hypofibrinolysis may be common features of DM and COVID-19 because of the hyperactivation of endothelial cells and changes in hemostatic factors [22, 25–27]. In this study, patients with DM had higher levels of fibrinogen. Deficiencies in procoagulant factors and fibrinolytic mechanisms are exacerbated when both DM and COVID-19 are present. The combination of original insufficient fibrinolysis in DM patients and severe COVID-19 alterations leads to a dramatic decrease in the ability of the body to dissolve blood clots, which manifests itself as elevated fibrinogen. Therefore, aggressive glycemic control and anticoagulation therapy as necessary can improve COVID-19 prognosis.

The COVID-19 usually presents as pneumonia, and those most severely affected will develop respiratory failure. Previous studies have shown that diabetic patients hospitalized for COVID-19 have a higher risk of respiratory failure, tracheal intubation, and mortality [2, 28]. It was interesting to note that we found no significant difference in the risk of respiratory failure, tracheal intubation, or death between the diabetic and nondiabetic groups. A study of lung tissue samples found no difference in ACE2 mRNA expression in the lungs of subjects with and without diabetes, despite elevated levels of ACE2 protein in alveolar tissue and bronchial epithelial cells in diabetic patients [29]. In addition, this may also be related to differences in novel coronavirus strains. Consistent with the results of two studies [30, 31], we found that diabetic patients had a higher mortality rate than non-diabetic patients, but the difference between the two groups was not statistically significant. This may be attributed to the fact that patients with T2DM may have more severe conditions and faster progression, leading to higher ICU admission rates. However, active treatment in the ICU (such as respiratory support, monitoring, and blood glucose management) effectively reduced their risk of death. Of course, it cannot be denied that due to cultural differences in China, some patients may choose to be discharged directly from the hospital at the end of their lives, which may lead to biased results.

Consistent with the results of other studies [32–34], we found that patients with T2DM had a higher rate of ICU demand, suggesting that COVID-19 patients with diabetes have worse clinical outcomes. In this study, we found that patients admitted to the ICU had worse inflammatory markers and coagulation, more severe hepatic impairment, myocardial injury, and malnutrition, all of which suggested impaired multiorgan function and a severely adverse prognosis for patients admitted to the ICU. Moreover, Neut and LDH were independent risk factors for ICU admission in patients with COVID-19 combined T2DM. LDH is an enzyme that indicates lysis of cells in different parenchymal organs such as the heart, liver, muscle, lungs, and bone marrow, and is a marker of inflammation as well as a predictor of pneumonia [35]. The severe COVID-19 combined with T2DM patients will result in high LDH levels due to their inflammatory lesions and cytolysis. It is well known that patients with severe COVID-19 have elevated leukocyte and neutrophil counts and significantly lower lymphocyte counts on admission, which correlates with the effects of viral infection on T cells via the ACE2 receptor [36]. As a result, both Neut and LDH suggest a more severe inflammatory response and poorer immune function in patients with COVID-19 combined T2DM, leading to an increased risk of ICU admission for them.

Although various prediction models have been constructed for COVID-19 patients admitted to the ICU, there is a lack of risk prediction models for diabetic patients admitted to the ICU. In this study, a binary logistic regression analysis was used to obtain a risk prediction model for patients with COVID-19 comorbid T2DM admitted to the ICU, and the predictive effect of the risk prediction model was examined by ROC curve analysis. The AUC was 0.80, and internal validation using bootstrapping showed negligible optimistic bias, both indicating that the model has good predictive ability. It provides an effective tool for the clinicians to evaluate the patients with high risk of admission to the ICU.

This study has the advantage of being a multicenter study with a sufficient sample size and a comprehensive clinical record. In addition, we not only analyzed the effect of DM on COVID-19, but also constructed a prediction model for the occurrence of adverse outcomes in COVID-19 combined with DM, which complemented the lack of this prediction model. Finally, there are some limitations of the present study. First, the current paper is a retrospective study, and the accuracy and reliability of the data from the medical record system phone may vary from individual to individual. Second, we did not analyze the effect of BMI and medication history on outcomes due to the high level of missing clinical data. Moreover, owing to the relatively small sample size of patients transferred to the ICU, the predictive model lacked external validation, which we will continue to complete by collecting data in a follow-up study.

## Conclusion

In conclusion, T2DM has resulted in significantly higher levels of certain inflammatory and biochemical markers, and greater susceptibility to coagulation abnormalities and deterioration of renal function in COVID-19 patients. In addition, DM increases the risk of ICU admission. Neut and LDH are risk factors predicting ICU transfer in patients of COVID-19 combined with T2DM. In this study, we constructed a prediction model for the ICU admission of COVID-19 patients with T2DM, and the predictive efficacy of the model was verified to be effective, which is of certain value for clinical application.

## Data Availability

The raw data supporting the conclusions of this article will be made available by the authors, without undue reservation.

## Abbreviations

ACE2: Angiotensin-converting enzyme 2
ALB: Albumin
ALT: Alanine aminotransferase
APTT: Activated partial thromboplastin time
AST: Aspartate aminotransferase
CAD: Coronary artery disease
CKD: Chronic kidney disease
CK-MB: Creatine kinase isoenzyme
COPD: Chronic obstructive pulmonary disease
COVID-19: Coronavirus disease 2019
Crea: Creatinine
CRP: C-reactive protein
DBIL: Direct bilirubin
DM: Diabetes mellitus
Fib: Fibrinogen
HGB: Hemoglobin
ICU: Intensive care unit
IL-6: Interleukin-6
K+: Potassium
LDH: Lactate dehydrogenase
Lymph: Lymphocyte count
Na+: Sodium
Neut: Neutrophil count
PCT: Procalcitonin
PLT: Blood platelet
PT: Prothrombin time
ROC: Receiver operating characteristic
SARS: Severe acute respiratory syndrome
SARS-CoV-2: Severe acute respiratory syndrome coronavirus 2
T2DM: Type 2 diabetes mellitus
TBIL: Total bilirubin
WBC: White blood cell count
